# Actein Inhibits Tumor Growth and Metastasis in HER2-Positive Breast Tumor Bearing Mice via Suppressing AKT/mTOR and Ras/Raf/MAPK Signaling Pathways

**DOI:** 10.3389/fonc.2020.00854

**Published:** 2020-05-27

**Authors:** Xiao-Xiao Wu, Grace Gar-Lee Yue, Jin-Run Dong, Christopher Wai-Kei Lam, Chun-Kwok Wong, Ming-Hua Qiu, Clara Bik-San Lau

**Affiliations:** ^1^Department of Chemical Pathology, The Chinese University of Hong Kong, Hong Kong, China; ^2^Institute of Chinese Medicine, The Chinese University of Hong Kong, Hong Kong, China; ^3^State Key Laboratory of Research on Bioactivities and Clinical Applications of Medicinal Plants, The Chinese University of Hong Kong, Hong Kong, China; ^4^State Key Laboratory of Phytochemistry and Plant Resources in West China, Kunming Institute of Botany, Chinese Academy of Sciences, Kunming, China; ^5^State Key Laboratory of Quality Research in Chinese Medicines, Faculty of Medicine, Macau University of Science and Technology, Macau, China; ^6^Li Dak Sum Yip Yio Chin R & D Centre for Chinese Medicine, The Chinese University of Hong Kong, Hong Kong, China

**Keywords:** actein, metastasis, brain-metastasis, human epidermal growth factor receptor 2 (HER2), HER2-positive breast cancer

## Abstract

HER2-positive breast cancer accounts for 15–20% in breast cancer and 50% of the metastatic HER2-positive breast cancer patients died of central nervous system progression. The present study investigated the effects of actein (a natural cycloartane triterpene) on cells adhesion, migration, proliferation and matrix degradation, and its underlying mechanism in HER2-positive breast cancer cells. The *in vivo* effect of actein on tumor growth and metastasis in MDA-MB-361 tumor-bearing mice as well as the anti-brain metastasis in tail vein injection mice model were also investigated. Our results showed that actein inhibited HER2-positive breast cancer cells viability, proliferation and migration. Actein also induced MDA-MB-361 cells G1 phase arrest and inhibited the expressions of cyclins and cyclin-dependent kinases. For intracellular mechanisms, actein inhibited the expressions of molecules in AKT/mTOR and Ras/Raf/MAPK signaling pathways. Furthermore, actein (15 mg/kg) was shown to exhibit anti-tumor and anti-metastatic activities in MDA-MB-361 breast tumor-bearing mice, and reduced brain metastasis in tail vein injection mice model. All these findings strongly suggested that actein is a potential anti-metastatic agent for HER2-positive breast cancer.

## Introduction

Breast cancer exhibits the highest incidence rate of cancers among women worldwide, in which 15–20% breast cancer presented with human epidermal growth factor receptor 2 (HER2) overexpression (1). Breast cancer with HER2 overexpression is defined by immunohistochemistry status (IHC3+) or fluorescence *in-situ* hybridization (FISH) measurement of a *HER2* gene copy number above six or a *HER2*/*CEP17* ratio equals or greater than two (2). Patients with HER2-positive breast cancer would have an increased aggressiveness, poor prognosis and short survival (3). Moreover, it has a higher probability of brain metastasis compared to the HER2-negative subtype (4). It has been reported that 6–16% of patients with breast cancer will develop brain metastasis (5), although autopsy data indicate that this rate may be as high as 36% (6, 7). Nowadays, the available treatments for HER2-positive breast cancer includes anti-HER2 agents targeting the HER2 family intracellularly or extracellularly, such as trastuzumab, lapatinib, pertuzumab, trastuzumab emtansine, and neratinib (1). The combination of pertuzumab, trastuzumab and docetaxel is the first-line clinical treatment for HER2-positive breast cancer (8), which could significantly prolong the progression-free and overall survival (9).

However, one of the major challenges in the treatment of HER2-positive metastatic breast cancer is brain metastases, in which a previous study demonstrated that up to 50% of the metastatic HER2-positive breast cancer patients eventually died of central nervous system (CNS) progression (10). This may due to the fact that trastuzumab and pertuzumab are unable to penetrate the blood-brain-barrier (BBB) because of the large molecular weight (11–13). Patients with metastatic HER2-positive breast cancer receiving front-line therapeutics have higher incidences of CNS metastases ranging from 28 to 43% (10) than those reported historically (9). In recent decades, a variety of HER2 inhibitors with small molecular weight have been developed and approved for relapsed HER2-positive breast cancer, including lapatinib (14) which can theoretically penetrate through the BBB. Nevertheless, emerging evidence indicates that the average distribution concentration of lapatinib in brain metastases was only 10–20% of the concentration found in peripheral metastases (15, 16), which can hardly treat the brain metastasis of HER2-positive breast cancer. Therefore, there is still an urgent need for a small molecular agent which can travel through BBB and possess anti-metastatic effect for HER2-positive breast cancer.

**Figure 1:**
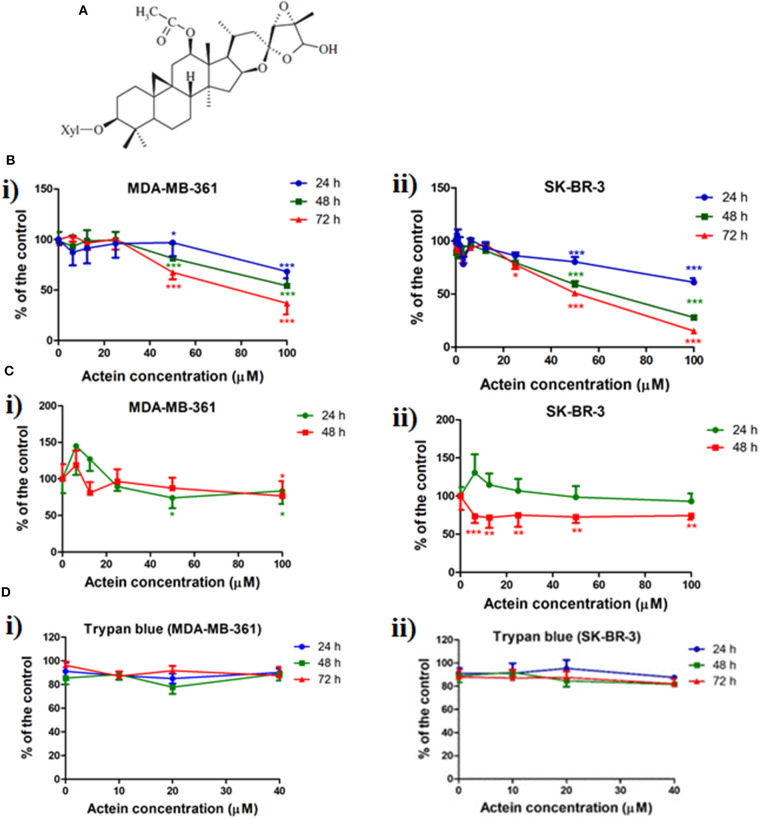
Chemical structure of actein and the inhibition effect of actein on cell viability and proliferation in HER2+ breast cancer cells. **(A)** Chemical structure of actein. **(B)** Cytotoxic effects of actein (6.25–100 μM) on (i) MDA-MB-361 and (ii) SK-BR-3 cells upon 24, 48, or 72 h treatment were performed using MTT assay. Data were expressed as the mean fold of untreated controls (mean ± SD of four independent experiments with five replicates each). **(C)** Effects of actein on (i) MDA-MB-361 and (ii) SK-BR-3 cells proliferation were investigated by (methyl-^3^H)-thymidine incorporation assay after 24 or 48 h incubation with actein. Results were expressed as mean ± SD of three independent experiments. **(D)** Trypan blue assays of actein (10–40 μM) on (i) MDA-MB-361 cells and (ii) SK-BR-3 cells (mean ± SD of three independent experiments with five wells each). Differences among the treated and vehicle treated control groups were determined by one-way ANOVA. *p < 0.05, **p < 0.01, and ***p < 0.001 as compared to control group.

Plant-derived compounds have long been recognized as valuable sources of clinically used anticancer drugs. Currently, several natural compounds have been approved by US Food and Drug Administration (FDA) for the treatment of breast cancer, such as vinblastine, docetaxel and paclitaxel (17). Actein (Figure 1A), is a natural cycloartane triterpenoid from roots of *Cimicifuga foetida* with a molecular weight of 676 g/mol. Several *in vitro* studies have demonstrated that actein exhibits inhibitory effects on a variety of cancers (18–21) including breast cancer (22–24). Actein exhibits the *in vitro* anti-proliferation and anti-migration effect in human non-small cell lung cancer cells (21) as well as osteosarcoma cells (25). For breast cancer, actein could inhibit the growth and proliferation of both mouse and human breast cancer cells (26, 27). Moreover, our previous studies have demonstrated that actein exhibited anti-angiogenic and anti-metastatic activities in mouse 4T1 mammary breast tumor-bearing model (26), and also the migration inhibition effect on human breast cancer xenograft zebrafish model (27).

The present study aimed to further investigate the effects of actein on tumor growth and metastasis in MDA-MB-361 (HER2-positive) tumor bearing mice, and also its effect on brain metastasis in SCID mice model (using tail vein injections of MDA-MB-361 cells). In addition, the effects of actein on cell proliferation, cell cycle phase distribution, cell migration and adhesion in HER2-positive breast cancer cells were also evaluated. Our results demonstrate the inhibitory effects of actein on tumor growth and metastasis in MDA-MB-361 tumor bearing mice model, as well as brain metastasis in breast cancer cells tail vein injections in SCID mice model. Actein was also shown to inhibit cell proliferation, migration and induce cell cycle arrest in HER2-positive breast cancer cells. The expression of phosphorylated proteins involved in protein kinase B (AKT)/mammalian target of rapamycin (mTOR) and rat sarcoma virus (Ras)/rapidly accelerated fibrosarcoma (Raf)/mitogen-activated protein kinase (MAPK) pathway of MDA-MB-361 cells were proven to be significantly reduced after actein treatment. Taken together, our findings suggested that actein has great potential to be developed as an anti-metastatic agent for the treatment of HER2-positive breast cancer.

## Materials and Methods

### Test Compound, Chemicals, and Reagents

Actein (Figure 1A) was extracted and isolated from roots of *Cimicifuga foetida*. The details of extraction and isolation procedures for actein have been reported previously (27). Actein (99% purity) in dry powder form was dissolved in dimethylsulfoxide (DMSO) at a concentration of 100 mM as the stock solution. The stock solution was stored at −20°C and reconstituted in appropriate medium prior to the experiments. DMSO (0.5% v/v) was used as the vehicle control.

The human epidermal growth factor receptor 2 (HER2) overexpression cell lines MDA-MB-361 and SK-BR-3 cells were purchased from American Type Culture Collection (Manassas, VA, USA). Leibovitz's L-15 medium, McCoy's 5A medium, fetal bovine serum (FBS), penicillin-streptomycin (PS) and trypsin-EDTA were obtained from Life Technologies (Grand Island, NY, USA). 3-(4,5-dimethylthiazol-2-yl)-2,5-diphenyl-tetrazolium bromide (MTT), propidium iodide (PI), RNase, and β-actin antibody were obtained from Sigma-Aldrich (St. Louis, MO, USA). Cell cycle related antibodies including polyclonal cyclin dependent kinase 2 (CDK2), monoclonal p21, monoclonal Cyclin E1 and polyclonal phosphorylated Rb (pRb) were purchased from Abcam (Cambridge, MA, USA). Anti-human polyclonal matrix metalloproteinase-2 (MMP-2), monoclonal matrix metalloproteinase-9 (MMP-9), polyclonal Ras, polyclonal Raf, monoclonal p-p38 MAPK, polyclonal p38 MAPK, polyclonal extracellular signal-regulated kinase (MEK), polyclonal Src, polyclonal AKT, monoclonal pAKT, monoclonal mTor and polyclonal pmTor antibodies were obtained from Cell Signaling Technology (Danvers, MA, USA). Rabbit anti-human polyclonal IgG and HRP-goat anti-mouse polyclonal IgG antibodies were obtained from Life Technologies (Grand Island, NY, USA). Transwell polycarbonate cell culture inserts (6.5 mm diameter, 8 μm pore size) were purchased from Corning (Lowell, ME, USA). [Methyl-^3^H]-thymidine was obtained from PerkinElmer (Waltham, MA, USA). Extracellular matrix cell adhesion array kit and uPA activity kit were purchased from Sigma-Aldrich (St. Louis, MO, USA).

Female NOD/SCID mice (6–8 weeks old) were obtained from and maintained in Laboratory Animal Services Center at the Chinese University of Hong Kong. Basement membrane matrix Matrigel with growth factor was purchased from Corning (Lowell, MA, USA). 17β-Estradiol pellet (60-days release) was obtained from Innovative Research of America (Sarasota, Florida, USA). Ki67 was purchased from Abcam (Cambridge, MA, USA). Cytokeratin 8 (CK8) and DAPI were obtained from Thermo Scientific (Waltham, MA, USA). Lactate dehydrogenase (LDH) and creatine kinase (CK) serum enzyme kits for murine study were obtained from Stanbio Laboratory (Boerne, TX, USA).

### Cell Culture

SK-BR-3 cells were maintained in McCoy's 5A medium supplemented with 10% (v/v) FBS and 1% (v/v) PS, incubated at 37°C in a humidified atmosphere containing 5% CO_2_. MDA-MB-361 cells were cultured in the medium of Leibovitz's L-15 with 20% (v/v) FBS and 1% (v/v) PS, incubated in a free gas exchange with atmospheric air incubator at 37°C.

### Cytotoxicity and Cell Proliferation Assay

The effects of cytotoxicity of actein on MDA-MB-361 and SK-BR-3 cells were assessed using MTT assay. Cells (5 × 10^4^/mL) were seeded in 96-well flat-bottom culture plate in 100 μL of medium and incubated at 37°C overnight. Then, ascending concentrations of actein in 100 μL of medium were added to the cells. Control wells were added with 100 μL vehicle solvent [0.05% (v/v) DMSO] in cell culture medium (L-15 medium for MDA-MB-361cells, McCoy's 5A medium for SK-BR-3 cells). Cell viability and proliferation were determined according to the procedures described previously (27). The effects of actein on the viable cell number were also determined by trypan blue exclusion assay. After treated with actein for 24, 48, or 72 h, cell suspension was collected and washed with PBS once, and viable cells were counted.

### Transwell Migration Assay

According to our previous study, the migration ability of cells was determined using a modified Boyden chamber in transwell migration assay (27). Briefly, MDA-MB-361 and SK-BR-3 cells (5 × 10^4^/mL) in 100 μL of medium with 1% (v/v) FBS and actein in 100 μL medium [1% (v/v) FBS] were added to the upper chamber of each transwell inserts with final concentration (10–40 μM) in duplicate. In the meantime, 500 μL of medium with 10% (v/v) FBS was added to the lower chamber. Cells were fixed with methanol for 3 min and stained with hematoxylin for 5 min after incubation for 5 h at 37°C. The cells were photographed, and the migrated cells were quantified by counting.

### Urokinase-Type Plasminogen Activator (uPA) Chromogenic Activity Assay

The activities of uPA of MDA-MB-361 and SK-BR-3 cells were assessed using uPA activity kit (28). Cells (5 × 10^4^/mL) were seeded in a 24-well plate overnight and treated with actein (10–40 μM) in 0.5 mL culture medium with 1% (v/v) FBS and incubated for 48 h. Conditional medium was collected and centrifuged at 1,000 × *g* for 10 min. Supernatants were collected and assay was carried out according to the procedures recommended by the manufacturer.

### Extracellular Matrix Cell (ECM) Adhesion Assay

ECM adhesion assay was performed to evaluate the cell adhesion ability of MDA-MB-361 cells to human ECM proteins. Each well of an eight-well strip was pre-coated with one of the seven different human ECM proteins (collagen I, collagen II, collagen IV, fibronectin, laminin, tenascin, or vitronectin) or bovine serum albumin (BSA) as negative control. The assay was carried out according to the procedures recommended by the manufacturer. Briefly, MDA-MB-361 cells were added to pre-coated wells treated with or without actein at 10–40 μM for 2 h. After washing several times with distilled water, stained conjugated cells were dissolved in extraction buffer. The plate was measured by a microplate reader (BioTek, USA) at 540 nm, and the change in optical density was represented as folds of untreated control.

### Cell Cycle Analysis

MDA-MB-361 cells and SK-BR-3 cells (3 × 10^5^/well) were seeded onto a 6-well plate with 3 mL medium and incubated overnight. After 24 h starvation in 1% (v/v) FBS in medium, medium containing 10% (v/v) FBS with actein (10–40 μM) were added into the wells and incubated for 24 or 48 h. Cells were washed with PBS containing 5% (v/v) FBS and ice-cold ethanol (70%) was used to permeabilize cell membrane at 4°C overnight. On the next day, cells were re-suspended in PBS containing RNase A (10 μg/mL) and propidium iodide (20 μg/mL) for 30 min in the dark at 37°C and then analyzed by FACSC Canto II flow cytometer (BD Biosciences, CA, USA).

### Western Blot Analysis

MDA-MB-361 cells (1 × 10^6^/well) were seeded in a 100 mm dish and incubated overnight to allow attachment. Fresh medium with actein (10–40 μM) was added to the cells and incubated for 24 or 48 h. Cells were harvested and lysed in lysis buffer and subjected to electrophoresis with 10% SDS-polyacrylamide gel and then transferred to a polyvinylidene difluoride membrane for protein expression analysis according to the previously described protocol (29). Briefly, blots were incubated overnight with primary antibodies against human β-actin, p21, CDK2, Cyclin E1, phosphorylated Rb, MMP-2, MMP-9, Ras, Raf, p-p38 MAPK, p38 MAPK, MEK, Src, AKT, pAKT, mTor and pmTor. After incubation with the secondary antibodies HRP-goat anti-rabbit IgG or HRP goat anti-mouse IgG for 1 h, the blots were detected using ECL solution and captured by a molecular imager, ChemiDoc XRSC (Bio-Rad Laboratory, Hercules, CA, USA). The bands intensities were quantified using ImageJ (NIH, USA). The intensities of bands were normalized to their own internal standard proteins (β-actin) for each protein samples. The quantitative data presented as fold of untreated control.

### MDA-MB-361 Breast Tumor-Bearing Mice Model

Female SCID mice (6–8 weeks old) were supplied and maintained by Laboratory Animal Service Center at the Chinese University of Hong Kong. Animal studies on actein were conducted using the MDA-MB-361 breast tumor-bearing mice model. All experimental procedures were approved by the Animal Experimentation Ethics Committee of The Chinese University of Hong Kong (Ref. No. 16/196/MIS). Female SCID mice were firstly implanted intradermally with 17β-estradiol pellet on the back, then the mixture of MDA-MB-361 cells (5 × 10^6^ cells in 50 μL PBS) and Matrigel in growth factor (50 μL) were subcutaneously inoculated at the mammary fat pad of each SCID mice on Day 0. There are two routes for actein administration according to previous studies, one is oral administration (30), the other is intraperitoneal (*i.p*.) injection (31), and both routes were applied in our *in vivo* studies. Two weeks later after inoculation, mice were randomized into four groups (*n* = 12). For oral administration, mice were treated with either vehicle, actein (5 or 15 mg/kg) or trastuzumab (10 mg/kg) (32) as positive control for 4 weeks. For *i.p*. injection, mice were administered with either PBS (control group), actein (15 or 20 mg/kg for first 10 days followed by 15 mg/kg for the remaining treatment period) or trastuzumab (15 mg/kg daily, once a week) (33) for 4 weeks. The doses of actein used in mice were chosen after the dose-finding pilot study (data not shown). During treatments, the body weight and tumor size of each mouse were measured twice a week. At the end of experiment, the mice were anesthetized, and the tumors, livers, brains and lungs were excised for further analysis. Also, plasma was collected for serum enzyme analysis including lactate dehydrogenase (LDH) and creatine kinase (CK).

### Tail Vein Injections in SCID Mice

Female SCID mice (6–8 weeks old) were obtained from and maintained in Laboratory Animal Services Center at the Chinese University of Hong Kong, and used for the study of brain metastasis following the injection of MDA-MB-361 cells into the tail vein of each mouse. Human breast cancer MDA-MB-361 cells (5 × 10^5^ in 100 μL PBS) were intravenously injected into the tail vein of each SCID mouse to allow breast cancer cells dissemination to the CNS (34). After 3 weeks, the animals were randomly divided into three groups (*n* = 6 in each group), and *i.p*. administration with PBS, actein (15 mg/kg), or trastuzumab (15 mg/kg, once per week) for 4 weeks. At the end of experiment, the mice were anesthetized, and the lungs and lymph nodes were removed and used for PCR analysis. Brains were also excised for immunohistochemistry staining, LCMS and PCR analyses.

### Plasma Enzyme Analysis

Plasma was collected by centrifugation of the blood (1,700 × *g*, 10 min, 4°C) and stored at −80°C until use. Concentrations of lactate dehydrogenase (LDH) for assessment of liver damage, and creatine kinase (CK) for assessment of heart damage, were analyzed using respective enzyme assay kits according to the procedures recommended by the manufacturer.

### Histological Processing and Immunohistochemical Analysis

Organs were dissected out after cervical dislocation and fixed in 10% formalin for 3 days prior to sample embedding. Hematoxylin and eosin (H&E) staining was performed on paraffin embedded lung and liver tissue sections (5 μm thick) to determine the cancer cells metastases in lungs and livers of mice, according to the procedures described previously (35). Tumor burden was calculated from the section of the lung or liver and expressed as an average percentage of tumor area to lung or liver area in each treatment group. Tumor sections were stained with Ki67 antibody as described previously (36). Immunoreactive species were detected using 3,3-diaminobenzidine tetrahydrochloride (DAB) as a substrate. Images were taken using an Olympus IX71 microscope and positive immunostaining cells in tumor sections was counted manually in a double-blind manner.

### Immunofluorescence Staining of Brain Tissue

Brain sections were stained with human cytokeratin 8 (CK8) antibody to identify human-derived epithelial tumor cells in brain (37). The expression of CK8 in brain sections further demonstrated how much breast cancer cell metastasis to mice brain. The more human breast cancer cells migrated to the mice brain, the stronger expression of CK8 in brain sections was detected. Brain sections (5 μm) were de-paraffinized in xylene and rehydrated in a series of graded alcohol. Then slides were subjected to an antigen retrieval buffer Tris-EDTA (pH = 9.0) and labeled with CK8 monoclonal antibody (2 μg/mL) in 0.1% BSA and incubated overnight at 4°C. The next day, slides were stained with secondary antibody at a dilution of 1:2,000 for 1 h at room temperature and nuclei were stained with DAPI for 5 min. The expression of CK8 was visualized on the sections, appeared as green in color under a fluorescent microscope Leica DMI6000 B (Leica Microsystems Ltd., Wetzlar, Germany).

### LCMS Analysis of Actein in Brain Tissue

To determine whether actein could penetrate through BBB, an Agilent 1290 UHPLC with 6530 QTOF LC/MS/MS system (CA, USA) was used, with an Agilent ZORBAX Eclipse Plus C18 RRHD (1.8 μm, 2.1 × 150 mm) column. Pure actein compound was dissolved in acetonitrile to give a concentration of 1 mg/mL and stored at −20°C until use. The stock solutions were extemporaneously diluted to 50 μg/mL as the standard working solutions. Standard working solution (10 μL) was spiked into one of the brains collected from vehicle-treated group, then extracted by below method which is similar to the brains in actein-treated group. Firstly, methanol (1 mL) was added to the brain and homogenized by ultrasonic probe, then centrifuged at 12,000 rpm for 10 min. Supernatant was removed and evaporated to dryness under nitrogen stream. The residue was re-dissolved in 50 μL methanol, vortexed and ultrasonicated for 5 min. Then, mixture was centrifuged at 12,000 rpm for 10 min, followed by transferring supernatant into glass insert and injecting 5 μL volume into the LCMS for analysis. The chromatographic separation was conducted at 40°C under gradient conditions at a flow rate of 0.5 mL/min. The LC profile was as follows: Mobile phase: (A) 0.1% formic acid in deionized water, and (B) 0.1% formic acid in methanol; Gradient: 0–3 min, 40–90% B. After each injection, column was flushed with 100% B for 1.5 min and re-equilibrated for another 3 min. The nebulizer had a pressure of 50 psig with a drying gas flow and temperature of 10 L/min and 350°C, respectively. Spectra were recorded in positive ion mode at spray voltage of 4,000 V. A targeted MS/MS was acquired at range 100–1,100 m/z. Actein was determined at 699.3733 m/z [M+Na]+.

### Real Time-PCR Analysis

Brains, lymph nodes and lungs excised from actein-treated or untreated MDA-MB-361 tail vein injections in SCID mice were snap frozen in liquid nitrogen. The total RNA of each organs was extracted and subjected to reverse transcription as described previously (36). To quantify the amount of human specific gene chromosome 17 (Cr17) (38), RT-PCR was performed in Bio-Rad CFX96™ Real-time system C1000 Thermal cycler using the QuantiFast SYBR Green RT-PCR kit from Qiagen (36). The sequences of Cr17 primer were: Forward primer GGGATAATTTCAGCTGACTAAACAGG; Reverse primer AAACGTCCACTTGCAGATTCTAG. The specific gene mRNA levels were normalized to that of the housekeeping gene GAPDH and then expressed as the fold change compared to the control group.

### Statistical Analysis

Data were expressed as mean + SD (*in vitro*) or mean + SEM (*in vivo*). Statistical analyses and significance were analyzed by one-way ANOVA with Tukey's *post-hoc* test using GraphPad PRISM software version 6.0 (GraphPad Software, CA, USA). In all comparisons, *p* < 0.05 was considered as statistically significant.

## Results

### Actein Inhibited Cell Viability and Cell Proliferation in MDA-MB-361 and SK-BR-3 Cells

Effect of actein (Figure 1A) on cell viability and cell proliferation in MDA-MB-361 and SK-BR-3 cells was determined by MTT and (methyl-^3^H)-thymidine incorporation assay. As shown in Figure 1B, actein (50–100 μM) inhibited cell viability in both two cell lines after treated for 24, 48, or 72 h. The concentration for 50% growth inhibition (IC_50_) of actein were listed in Table 1, all were above 50 μM. Besides, cell proliferation was significantly inhibited by actein at 100 μM after 48 h treatment in both cell lines (Figure 1C). Since our purpose was to investigate the anti-metastatic effect of actein on breast cancer cells without inducing high cytotoxicity, low concentrations of actein were applied in the subsequent culture assays. Results of MTT assay showed that actein (3.25–40 μM) did not exert any significant cytotoxicity in MDA-MB-361 and SK-BR-3 cells (with all *p* > 0.05, Figure 1B). Trypan blue assay results also showed that actein (10–40 μM) did not affect the viability of MDA-MB-361 and SK-BR-3 cells (Figure 1D). Therefore, actein at up to 40 μM was applied in the subsequent cell assays on both cell lines.

**Table 1:** A list of IC_50_ values of actein on MDA-MB-361 and SK-BR-3 cells.

**IC_**50**_ (μM)**	**24 h**	**48 h**	**72 h**
MDA-MB-361	>100	>100	70.8
SK-BR-3	>100	64.9	52.6

### Actein Inhibited Cell Migration in Human Breast Cancer Cells

**Figure 2:**
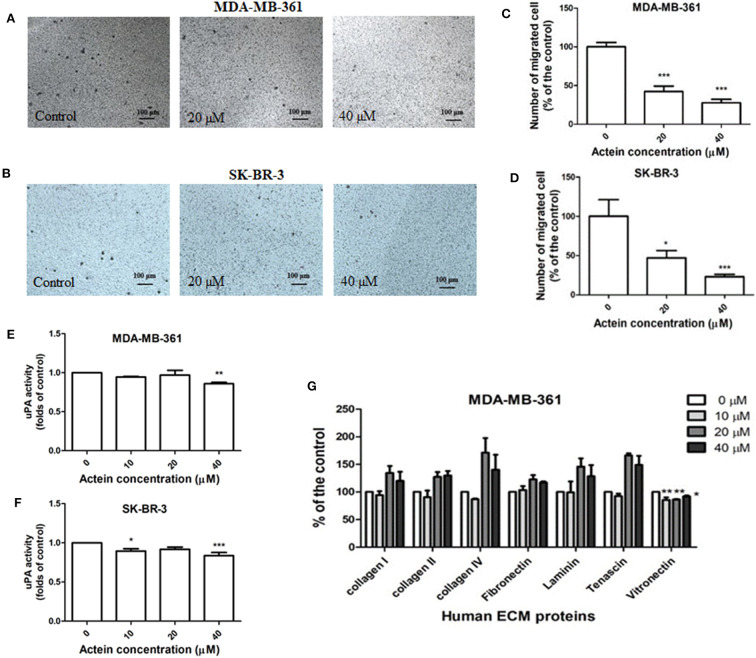
Actein inhibited cell migration in HER2+ breast cancer cells. Cells were treated with actein for 5 h in transwell migration assay. Representative photographs show the stained migrated **(A)** MDA-MB-361 cells or **(B)** SK-BR-3 cells on the lower side of the membrane after incubation. **(C,D)** Quantitative analysis summarized the number of migrated cells on the lower chambers (mean + SD of three independent experiments with duplicates each) and expressed as the percentage of the control. **(E,F)** uPA activity assay. uPA activity was measured using a specific uPA substrate releasing a colored chromophore (mean + SD of eight independent experiments in duplicate). **(G)** Inhibitory effect on MDA-MB-361 adhesion to ECM proteins by ECM-adhesion assay. Results were expressed as the mean fold of the untreated control (mean + SD of three independent experiments in duplicates). Statistical differences were determined by One-way ANOVA, with *p < 0.05, **p < 0.01, ***p < 0.001 against untreated controls.

The effects of actein on the migration of breast cancer cells were evaluated in modified Boyden chambers. MDA-MB-361 and SK-BR-3 cells could migrate across the pore of the membrane from the upper chamber to the lower chamber. Representative photographs of the stained migrated cells on the lower side of the membrane are presented in Figures 2A,B. In the presence of actein (20 and 40 μM), the number of migrated MDA-MB-361 cells were significantly decreased by 57 and 72%, respectively (Figure 2C). For SK-BR-3 cells, actein also inhibited about 53 and 77% migrated cells compared to the control wells, at 20 and 40 μM, respectively (Figure 2D), thereby suggesting the inhibitory effect of actein on the migration of HER2-positive human breast cancer cells.

uPA is ECM-associated proteinases involved in matrix degradation. Actein (40 μM) slightly attenuated the activity of uPA secreted by MDA-MB-361 and SK-BR-3 cells after 24 h incubation (Figures 2E,F). ECM adhesion assay showed that actein (10–40 μM) significantly inhibited MDA-MB-361 cell adhesion to vitronectin (Figure 2G). However, adhesion capabilities were enhanced in other human ECM proteins including collagen I, collagen II, collagen IV, laminin and tenascin.

### Actein Induced G1 Phase Arrest in Human Breast Cancer Cells

**Figure 3:**
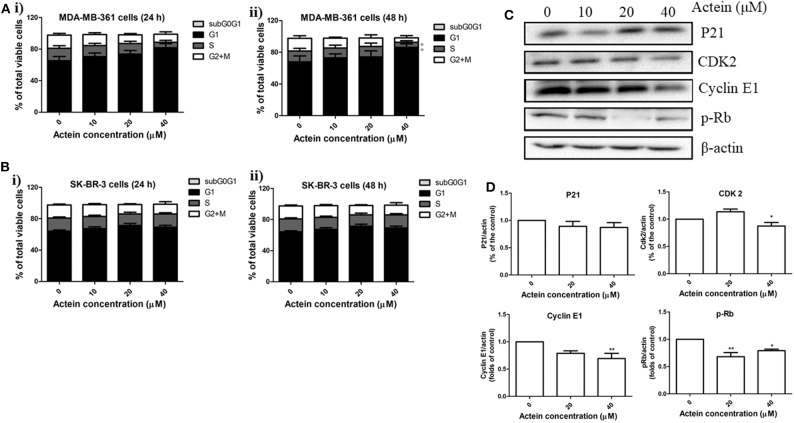
Actein mediated MDA-MB-361 cell arrest in G1 phase through the modulation of p21-cyclin E1/CDK2-pRb pathway. Cell cycle of **(A)** MDA-MB-361 cells and **(B)** SK-BR-3 cells treated with actein for (i) 24 and (ii) 48 h were evaluated by flow cytometry, results were presented as mean + SD of four independent experiments in bar charts. **(C)** Western blot analysis of p21-cyclin E1/CDK2-pRb pathway related proteins in MDA-MB-361 cells upon 48 h treatment with actein (10–40 μM). **(D)** Bar charts show the expression levels of proteins of P21, CDK2, Cyclin E1, and pRb, which were adjusted with corresponding β-actin protein level and expressed as fold of control (mean fold of control + SD from four independent experiments). Differences among the treated and vehicle treated control groups were determined by One-way ANOVA. *p < 0.05 and **p < 0.01 as compared to control group.

Effects of actein on cell cycle distribution were analyzed by flow cytometry after MDA-MB-361 cells and SK-BR-3 cells treated with actein for 24 or 48 h (Figures 3A,B). Results demonstrated that actein significantly increased the percentage of cells in G1 phase with a corresponding decrease in the S phase in MDA-MB-361 cells in 48 h incubation. However, there was no significant effect of actein on the cell distribution in SK-BR-3 cells at both 24 and 48 h treatment. Western blot experiment was performed on 48-h time point to further investigate the expressions of cyclins and cyclin-dependent kinases during G1 phase arrest. As shown in Figure 3C, the expressions of CDK2, Cyclin E1 and p-Rb of MDA-MB-361 cells were significantly inhibited by actein (20–40 μM), while the expression of p21 remains unchanged (Figure 3D). This might be associated with G1 phase arrest induced by actein.

### Effects of Actein on the Expression of Proteins in Various Signaling Pathways

**Figure 4:**
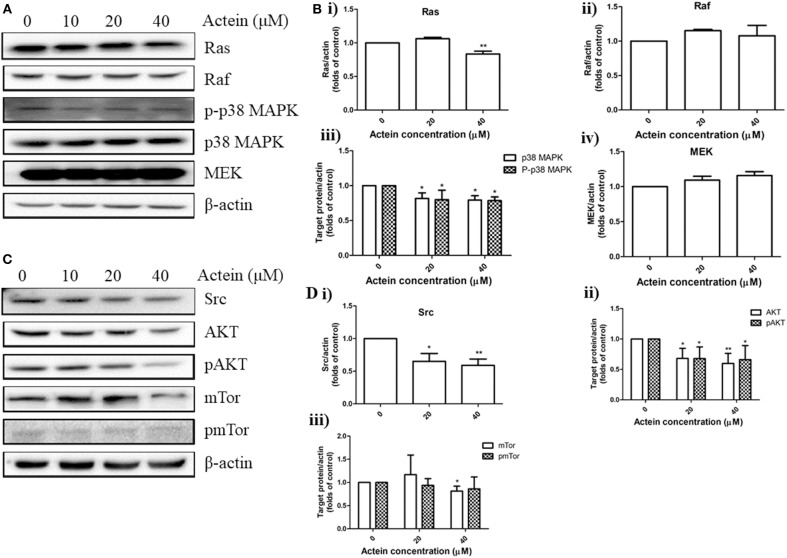
Effects of actein on the expression of proteins in various signaling pathways in MDA-MB-361 cells. **(A,C)** Representative Western blots showing the expressions of Ras/Raf/MAPK and AKT/mTor signaling pathways related proteins in MDA-MB-361 cells. **(B,D)** Bar charts showing the results of the protein expression of Ras, Raf, p38MAPK, pp38MAPK, Src, AKT, pAKT, mTor, and pmTor using Western blot analysis, which were normalized with corresponding β-actin protein expression and expressed as fold of control (mean + SD of four independent experiments). Differences among the treated and vehicle treated control groups were determined by One-way ANOVA. *p < 0.05 and **p < 0.01 as compared to control group.

Accumulating evidences suggested the HER2-mediated signaling pathways play a critical role in breast cancer metastasis (39). Our results above had already indicated the anti-migration of actein in HER2-positive breast cancer cells. Here, we further investigated whether actein exhibited its anti-metastatic effect through the modulation of HER2-related signaling pathways in MDA-MB-361 cells. Western blot assay was performed to test the *in vitro* effect of actein on the pathways of AKT/mTOR and Ras/Raf/MAPK in MDA-MB-361 cells (Figures 4A,C). Actein significantly inhibited the expression of Ras, p38MAPK and p-p38MAPK after 24-h treatment, without any significant effect on Raf and MEK expression (Figure 4B). Moreover, expressions of Src, AKT, pAKT and mTor were significantly inhibited by actein (20–40 μM) while no significant effect on pmTor (Figure 4D). Taken together, actein might have exerted its anti-metastatic activity through inhibiting AKT/mTOR and Ras/Raf/MAPK pathways in MDA-MB-361 cells.

### Orally Administered Actein Suppressed the Metastasis of Breast Cancer to Lung and Liver in SCID MDA-MB-361 Tumor-Bearing Mice

**Figure 5:**
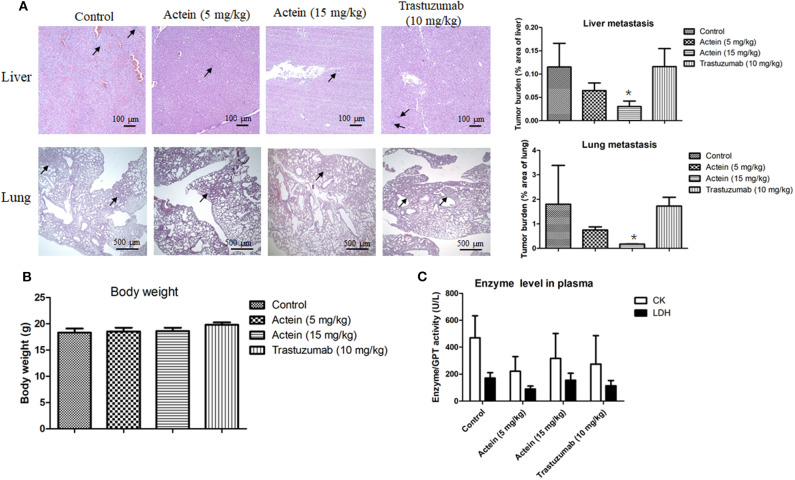
Histopathology of lungs and livers of tumor-bearing mice after actein oral administration. **(A)** The paraffin-embedded sections of the lungs and livers were photographed and used to measure metastatic loci area and total lung or liver area. The histograms showed the tumor burden in lungs and livers according to the tumor area as a percentage of whole lung or liver area per group. Representative H&E-stained sections of lungs and livers from different groups with arrows showing the metastatic loci. Results were expressed as mean + SD of 12 mice each group. **(B)** The final body weights of mice before sacrifice. **(C)** Plasma enzyme activities of LDH and CK were calculated at the end of the experiment. Differences among the treated and vehicle treated control groups were determined by One-way ANOVA. *p < 0.05 as compared to control group.

MDA-MB-361 mammary tumors were formed at the mammary fat pad of SCID mice in order to investigate the *in vivo* anti-metastatic activities of actein. Tumor-bearing mice were randomly divided into 3 groups of 12 mice each, and then orally administered with actein at 5 mg/kg per day, actein at 15 mg/kg per day, or trastuzumab *i.p*. administered at 10 mg/kg per day for 4 weeks. The anti-metastatic activities of actein were observed in lungs and livers of tumor-bearing mice treated with actein. Parafilm-embedded sections of lungs and livers were assessed for the tumor burden in a blinded manner. As shown in Figure 5A, large metastatic loci were found in vehicle-treated control group as well as the trastuzumab-treated group (positive control group), while the area of metastatic loci was decreased in actein-treated group. Tumor burden in lungs and livers from 5 mg/kg actein-treated group was found to decrease by 58.3 and 43.9%, respectively, when compared with vehicle-treated group (Figure 5A). Moreover, actein (15 mg/kg) significantly reduced lung and liver metastasis by 90.2 and 73.5%, respectively. Additionally, there was significant decrease of tumor cell metastasis in lungs and livers by 89.8 and 73.7%, respectively, when actein-treated group was compared to trastuzumab-treated group. There was no obvious body weight loss (Figure 5B) and no alteration of the plasma enzymes activities (CK and LDH) (Figure 5C) were observed in actein-treated group, suggesting that actein (15 mg/kg) oral administration could inhibit liver and lung metastasis in xenograft mouse breast tumor model without any observable toxicity.

### *In vivo* Anti-tumor Activities of Intraperitoneal Injection of Actein in Breast Tumor-Bearing Mice

**Figure 6:**
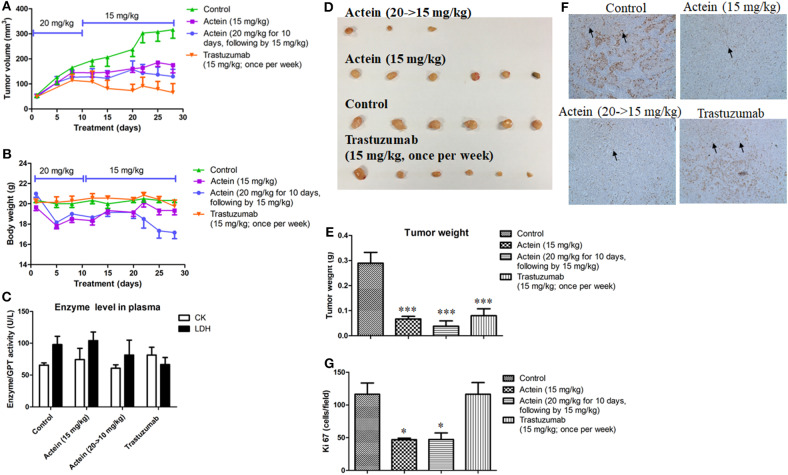
Anti-tumor effect of actein on MDA-MB-361 tumor-bearing mice. Mice were *i.p*. administered with actein or trastuzumab or vehicle for 4 weeks. The volumes of **(A)** tumors and **(B)** body weights were recorded and expressed as mean ± SEM (*n* = 12). **(C)** Plasma enzymes activities of LDH and CK were calculated at the end of the experiment. **(D)** The photos of tumors excised from mice of different treatment groups at the end of experiment. **(E)** Tumor weights were measured and expressed as mean + SEM. **(F)** Tumor tissues were examined by IHC staining with antibodies against Ki67 (brown staining). **(G)** Results showed the percentages of the Ki67 positive cells in the total number of cells per section. Each value was presented as means + SD (*n* = 4). *p < 0.05, ***p < 0.001, compared with control using One-way ANOVA.

Apart from oral administration, MDA-MB-361 tumor-bearing SCID mice were also intraperitoneally (*i.p*.) injected with actein for 4 weeks: the low dosage actein-treated group was *i.p*. injected with actein at 15 mg/kg per day, the high dosage actein-treated group was *i.p*. administered with actein at 20 mg/kg for the first 10 days followed by 15 mg/kg for the remaining 18 days. The positive control group was *i.p*. injected with trastuzumab at 15 mg/kg once per week for four times. The tumor volumes of actein-treated mice as well as trastuzumab-treated mice were significantly lower than those of untreated control mice since the 15th day of treatment (*p* < 0.05, Figure 6A). During the treatment, body weight loss was observed in the high dosage actein-treated group while there was no difference among low dosage actein-treated group and vehicle control group (Figure 6B). Moreover, high dosage of actein resulted in half of the mice died before the sacrifice day which means high dosage actein is toxic to mice, although there is no difference in plasma enzymes analysis results (Figure 6C). From these results, we could conclude that the tolerable dose of actein for *i.p*. injection in mice was 15 mg/kg. At the end of experiment, excised tumors were weighed as shown in Figure 6D. The tumor weights were significantly decreased by 75.5 and 56.9% after treated with high and low dosages of actein when compared with control group, respectively (Figure 6E). Trastuzumab also reduced tumor weight by 65.5%. To further verify our *in vitro* results that actein inhibited cell proliferation, the tumors sections were stained with proliferation marker Ki67 using immunohistochemistry method (Figure 6F). Expression of Ki67 was significantly decreased after actein but not trastuzumab treatment (Figure 6G).

### Actein Decreased Brain Metastasis of Breast Cancer Cells in Brain Metastasis Mouse Model

**Figure 7:**
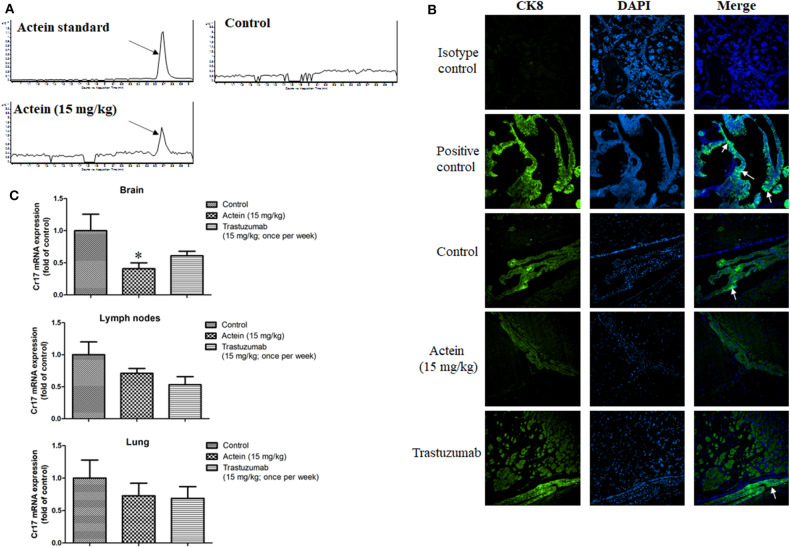
Actein suppressed brain metastasis in MDA-MB-361 cells tail vein injection in SCID mice. **(A)** LCMS results of actein in brain tissue with arrows showing the peak of actein. **(B)** Representative images of CK8 staining of brain tissues, arrows showing the metastatic loci. **(C)** Quantitative RT-PCR analyses of Cr17 gene in brain, lymph nodes and lungs. *p < 0.05 compared with control using One-way ANOVA.

We next examined the effect of actein on brain metastasis using a brain metastasis mouse model. LCMS results showed that brain in actein-treated group has the same peak as the actein standard while the brain from vehicle-treated group has no such peak, suggesting that actein could successfully cross through BBB (Figure 7A). As shown in Figure 7B, there is no signal of CK8 observed in the isotype control group. On the contrary, expression of CK8 in the positive control tissue was potent. For our mice brain sections, human-derived epithelial tumor cells were successfully identified in the groups treated with vehicle or trastuzumab, while in the actein-treated group, we can seldom find the positive stained cells in brain sections. This result suggested that actein could reduce MDA-MB-361 cells metastasis to brain. Additionally, the mRNA expression of human specific gene Cr17 in brain tissue was also examined using RT-PCR analysis. Actein (15 mg/kg) significantly downregulated the Cr17 expression by 59.1% when compared with control group (Figure 7C), while trastuzumab also downregulated the Cr17 expression by 39.0% compared to the vehicle group, but no obvious differences were observed in the lymph nodes and lung.

## Discussion

Compared to the normal cells, breast cancer cells have uncontrolled proliferation resulting in severe metastasis at different organs including bones, lungs and brain (40–42). Patients with metastatic HER2-positive breast cancer are at a high risk of developing parenchymal brain metastases (43) which is a major problem in the treatment of HER2-positive breast cancer. In the present study, we reported that actein at non-toxic dose exhibited anti-metastatic effect and inhibited the tumor growth on human breast tumor bearing mice, blocked tumor cells to brain-metastasis in MDA-MB-361 cells tail vein injection in SCID mice model. Furthermore, actein exerted significant inhibitory effect on HER2-positive breast cancer cells by decreasing cell viability, proliferation and migration *in vitro*. Moreover, actein caused cell cycle arrest at G1 phase as well as inhibited the expression of proteins in AKT/mTOR and Ras/Raf/MAPK signaling pathways of MDA-MB-361 cells.

MDA-MB-361 and SK-BR-3 cells, the commonly used HER2-positive breast cancer cell models, were applied in the present investigation (44). Herein, we reported that actein inhibited MDA-MB-361 and SK-BR-3 cell viability and cell proliferation. Previous studies had reported that the inhibition of cell cycle progression is capable of suppressing cell proliferation and blocking metastasis (45). Flow cytometry results demonstrated that actein suppressed cell cycle progression from G1 to S phase in MDA-MB-361 cells after treatment with actein for 48 h (Figure 3A). To further elucidate the underlying mechanism of the cell cycle arrest induced by actein, the expressions of pRb and the corresponding inhibitory cyclins and CDKs were observed. From our Western blot result, the downregulation of pRb, CDK2 and Cyclin E1 expression were observed when treated with actein for 48 h, while the expression of p21 remains unchanged (Figure 3D). Our findings suggested that actein most likely interfere with this p21-cyclin E1/CDK2-pRb pathway and consequently inhibit DNA synthesis and cell cycle progression (46). Results of transwell migration assay demonstrated that actein significantly inhibited MDA-MB-361 and SK-BR-3 cell migration in a dose-dependent manner. Actein also significantly inhibited MDA-MB-361 cell adhesion to vitronectin which serves to regulate proteolysis initiated by plasminogen activation and affects cell adhesion and motility (47). However, breast cancer cell adhesion to other human ECM proteins was enhanced. Therefore, the effect of actein on cell adhesion needs further investigation.

Breast cancer cells usually secrete matrix-associated proteases, such as matrix metalloproteinases (MMPs) and urokinase activator of plasminogen (uPA) to degrade the ECM which means attenuating the expression and activity of MMPs (48) and uPA (49) is also responsible for inhibiting breast cancer cell invasion and metastasis (45). Specifically, uPA converts the proenzyme plasminogen to the active form of plasmin, leading to the activation of MMPs involved in ECM remodeling and degradation, contributes to the breast cancer cell invasion and migration into the circulation (50). In the present study, western blot and uPA activity assay were performed to evaluate the effect of actein on MMPs and uPA. Results showed that actein has no significant effect on the activities of MMP-9 and MMP-2 of MDA-MB-361 as well as SK-BR-3 cells (data not shown), however, actein could slightly inhibit the activities of uPA secreted both by MDA-MB-361 and SK-BR-3 cells when treated with actein (40 μM) for at least 48 h (Figures 2E,F).

PI3K/AKT/mTOR and Ras/Raf/MAPK signaling pathways could regulate HER2 (51), in turn, HER2 overexpression will lead to increased expression of both the PI3K/Akt/mTOR and Ras/Raf/MAPK pathways (52). Pathogenesis of breast cancer and drug resistance in HER2-positive breast cancer brain metastasis appears partly to be driven by the activation of PI3K/AKT/mTOR pathway, suggesting that targeted inhibition of individual components in this pathway, including Akt and mTOR may be a potential strategy for HER2 positive breast cancer therapy (53, 54). Western blots results showed that actein significantly inhibited the expressions of AKT, pAKT and mTor in MDA-MB-361 cells. Additionally, expressions of Ras, p38MAPK and pp38MAPK which are the key downstream components of Ras/Raf/MAPK signaling pathways were also significantly reduced by the treatment of actein (Figure 4B). Taken together, actein may inhibit the metastasis of HER2-positive cancer cells through the AKT/mTOR or Ras/Raf/MAPK signaling pathways.

Furthermore, the *in vivo* anti-metastasis and anti-tumor effects of actein were confirmed using MDA-MB-361 tumor-bearing mice model, in which tumor cell metastasis to liver and lung were significantly reduced after actein (15 mg/kg) orally administered without any observable toxicity in mice. However, there is no significant effect on the tumor growth after actein oral administration. On the contrary, *i.p*. administration of actein (15 mg/kg) could significantly suppress tumor growth without any change on body weights and no alteration of the plasma enzymes activities while liver and lungs metastasis remain unchanged. Taken together, treatment with actein (15 mg/kg) by both oral and *i.p*. administration routes exhibited no toxicity in mice. However, toxicity in mice was observed upon *i.p*. injection with actein (20 mg/kg) for 10 days. Therefore, the tolerable dose of actein for *i.p*. injection in mice was 15 mg/kg, and the same applied in oral administration. As expected, consistent with our *in vitro* results, immunohistochemical analysis of Ki67 revealed that actein treatment inhibited tumor cell proliferation which may partly explain the anti-tumor activity of actein *in vivo*. This demonstrated that actein could effectively suppress tumor growth as well as metastasis *in vivo*. The different results between oral route and *i.p*. administration may be due to the differences in the bioavailability of actein with different administration (55), and it needs further experiments to confirm. Compared to trastuzumab (first line chemotherapeutics agent used in HER2-positive breast cancer), regarding to the tumor cell metastasis to liver and lung, trastuzumab had no significant inhibitory effect on breast cancer metastasis in MDA-MB-361 tumor bearing mice model. Additionally, results from immunohistochemical staining revealed that there was no obvious effect on the expression of Ki67 in tumor tissues after trastuzumab treatment.

The brain metastatic process consists of a series of steps. The primary tumor has to establish a blood supply in the breast and later provide an escape route for the primary breast tumor cells, then breast cancer cells must enter the circulatory system and survive in the blood circulation until they reach the brain (56–58). It takes a long time for the brain metastasis to be observed in our subcutaneously inoculated tumor-bearing mice model as brain metastasis typically occurs late in metastatic breast cancer, usually with preceding lung, liver or bone metastasis (59). Therefore, we established another model in which MDA-MB-361 cells tail vein injection directly in mice to study the anti-brain metastasis effect of actein (60). Through our pilot study, brain metastasis was observed in mice at 3 weeks later of breast cancer cells tail vein injection in each mouse. Mice were *i.p*. injected with actein (15 mg/kg) which was expected to be distributed in the whole body, including brain. LCMS results verified that actein could successfully penetrate through the BBB (Figure 7A). There are several factors affecting the ability of compounds that can cross the BBB, including lipid solubility, charge, tertiary structure and degree of protein binding. However, the main factor among all these is molecular weight (61). Compounds with low molecular weight and lipophilic properties can easily cross the BBB by simple diffusion (62), and usually the cut-off molecular weight is 400–600 g/mol. However, previous studies found that peptides or proteins with molecular weights over 600 g/mol are also able to cross the BBB (61). Therefore, the reason that actein could successfully cross the BBB is not only due to the molecular weight of actein, but also other collective effects which needs further investigation. The human-derived epithelial tumor cells CK8 staining showed that tumor cell loci in brains in the actein-treated group were decreased compared to the control group, while trastuzumab seems to have no effect on breast cancer cells metastasis to the brain. Additionally, compared to the control group, actein (15 mg/kg) significantly suppressed mRNA expressions of human specific gene Cr17 in mouse brain which means MDA-MB-361 cells metastasis to brain were reduced while trastuzumab has no significant effect on the mRNA expression of Cr17. However, sensitive diagnostic imaging tools, such as advanced computerized tomography (CT), magnetic resonance imaging (MRI) and positron emission tomography (PET) needs to be performed in mice with brain metastasis in further study (63).

Taken together, we showed that actein exhibited significant anti-metastasis activity through the modulation of AKT/mTOR and Ras/Raf/MAPK signaling pathway in HER2-positive breast cancer cells *in vitro*. Besides, our *in vivo* studies suggested that actein (15 mg/kg) treatment exerted anti-tumor and anti-metastatic effects in tumor bearing mice model, and also reduced brain metastasis in mice. It has great potential to be developed as a drug for the treatment of brain metastasis in HER2- positive breast cancer.

## Data Availability Statement

The original contributions presented in the study are publicly available. This data can be found here: Figshare with accession number doi: 10.6084/m9.figshare.12212642.

## Ethics Statement

This study was carried out in accordance with the guidelines of laboratory animal care and the experimental protocols have been approved by the Animal Experimentation Ethics Committee of the Chinese University of Hong Kong (Ref no. 16/196/MIS).

## Author Contributions

X-XW, GY, CB-SL, and C-KW designed the study. X-XW and GY performed the biological research and analyzed the data. J-RD and M-HQ extracted the chemical material and performed the chemical analysis. CB-SL, M-HQ, and C-KW contributed essential reagents and research facilities. X-XW wrote the manuscript. GY, CB-SL, CW-KL, and C-KW revised the manuscript. All authors reviewed the manuscript.

## Conflict of Interest

The authors declare that the research was conducted in the absence of any commercial or financial relationships that could be construed as a potential conflict of interest.
